# Editorial: Cognition and brain activity in Latin America

**DOI:** 10.3389/fpsyg.2024.1491240

**Published:** 2024-09-18

**Authors:** Alberto Rodríguez-Lorenzana, Guido Mascialino

**Affiliations:** ^1^Departamento de Ciencias de la Salud, Universidad Pública de Navarra, Pamplona, Spain; ^2^Escuela de Psicología y Educación, Universidad de Las Américas, Quito, Ecuador

**Keywords:** cognition, neuropsychology, executive function, Latin America, brain activity

In recent decades, the study of cognitive functions and their relationship with brain activity has experienced remarkable progress worldwide, especially in developed countries. This progress has meant a significant increase in the quantity and quality of published research, particularly in Europe and North America (Gazzaniga, 2018). In Latin America however, this development has been considerably slower. Recent data show that the number of studies exploring this topic in the region is substantially lower than those conducted in other parts of the world (Ardila, 2018).

There are likely multiple reasons for this disparity, including limited research funding, a shortage of trained professionals, fewer opportunities for advanced education and research, as well as linguistic and cultural differences that can isolate Latin American researchers from the global scientific community (Ciocca and Delgado, 2017; Vasconcelos et al., 2008). Despite this, the last decade has seen a considerable increase in the scientific production of the study of cognition in Latin America. This trend is due to greater participation of the region in the global landscape of neuropsychological research. It is crucial, therefore, to increase the visibility of these emerging works.

The aim of this Research Topic: “*Cognition and brain activity in Latin America*”, published in Frontiers in Psychology, is to highlight recent advances in the study of cognitive functions and their relationship with brain activity in Latin America, as well as identifying work teams exploring these areas in the region, creating new possibilities for future studies. This Research Topic brought together five original contributions.

In the opening article of this Research Topic, Vásquez-Pinto et al. explore the phenomenon of insight during problem-solving through the lens of dynamic systems theory, which posits that insight emerges from the self-organization of behavior and cognition in response to environmental information and uncertainty. The study focuses on the pupillary responses of children aged 6–12 as they engaged in the 8-coin task, utilizing Recurrence Quantification Analysis and Power Spectrum Density methods. The findings reveal that children who successfully solved the task exhibited higher entropy and lower determinism in their pupil diameter fluctuations prior to achieving insight, indicating a transition from a pre-insight state to the moment of insight. These results suggest that physiological markers like pupillary fluctuations may serve as early indicators of problem-solving success. Moreover, this Research Topic contributes to our understanding of cognition and brain activity within the Latin American context by demonstrating the value of advanced non-linear dynamics techniques in studying cognitive processes across diverse populations.

The article titled by Ramos-Galarza et al. presents the development and validation of a psychometric instrument aimed at assessing executive functions in university students from Chile and Ecuador. Executive functions, including conscious monitoring of behavior, decision-making, and emotional regulation, play a crucial role in academic success (Ahmed et al., 2019). The scale, UEF-1, was tested with a sample of 1,373 students and showed strong reliability and validity, through item correlation and confirmatory factor analysis, across seven key areas of executive functioning. This scale offers a valuable tool for research and intervention in Latin American higher education, addressing a previously underexplored population.

The third article by Mantilla and Ortiz-Merchán explores the relationship between strategic decision-making in cognitive hierarchy games and academic outcomes. Using the Lowest Unique Positive Integer (LUPI) game the authors assessed university students' abilities to engage in analytical reasoning and correlated this with short- and medium-term academic performance, as well as cognitive reflection. The study found that students who performed better in LUPI were more likely to achieve higher exam grades, course grades, and GPAs. Furthermore, students who performed better on the LUPI also demonstrated stronger analytical skills, as indicated by higher Cognitive Reflection Test scores. This Research Topic highlights the relevance of cognitive games in understanding strategic thinking as well as its relationship with academic achievement. Likewise, this study suggests the potential use of cognitive games as tools to enhance critical thinking in educational settings.

The fourth article (Jara-Rizzo et al.) explores through two studies the influence of different cognitive domains on biosecurity measure compliance during the pandemic. The results showed that cognitive control and beliefs about the severity of COVID-19 were significant predictors of compliance, while cognitive flexibility and working memory did not provide significant improvements in the predictive ability of the models. The findings reported by the authors underline the importance of considering cognitive and psychological factors in the design of interventions aimed at improving adherence to public health measures during health emergencies.

The last paper (Rodas et al.) explored the influence of executive functions (FFEE) on the ability to regulate emotion, paying special attention to negative emotions. The results reveal that the ability to switch task or focus is the only significant predictor of effective emotion regulation, while working memory and inhibitory control show no relevant impact. These findings highlight the determinant role that cognitive flexibility plays in emotion regulation, thus challenging the traditional notion that all FFEE are equally important in this process and pointing to the need for further research in this area.

This Research Topic highlights the role of understanding brain functioning and cognitive processes in the unique contexts of Latin American populations. The studies presented offer valuable insights that not only enrich our understanding of neuropsychological research in the region but also contribute to the global body of knowledge. These works also underscore the need for continued work in this area and region, while demonstrating how findings from Latin America can inform practices worldwide. We extend our deepest gratitude to the authors for their valuable contributions which will help further stimulate work in these areas.
